# Factors Affecting Food Consumers’ Behavior during COVID-19 in Romania

**DOI:** 10.3390/foods11152275

**Published:** 2022-07-29

**Authors:** Iulia C. Muresan, Rezhen Harun, Anca Monica Brata, Vlad Dumitru Brata, Daniel I. Chiciudean, Olivia Paula Tirpe, Andra Porutiu, Diana E. Dumitras

**Affiliations:** 1 Department of Economic Sciences, University of Agricultural Sciences and Veterinary Medicine Cluj-Napoca, 3-5 Manastur Street, 400372 Cluj-Napoca, Romania; iulia.muresan@usamvcluj.ro (I.C.M.); daniel.chiciudean@usamvcluj.ro (D.I.C.); ddumitras@usamvcluj.ro (D.E.D.); 2 Department of Agribusiness and Rural Development, College of Agricultural Sciences Engineering, University of Sulaimani, Sulaimani 5100, Iraq; 3 Department of Engineering of Food Products, Faculty of Environmental Protection, University of Oradea, 26 Gen. Magheru St., 410087 Oradea, Romania; abrata@uoradea.ro; 4 Faculty of Medicine, “Iuliu Hatieganu” University of Medicine and Pharmacy, 400000 Cluj-Napoca, Romania; brata_vlad@yahoo.com; 5 Department of Animal Husbandry and Agritourism, Faculty of Environmental Protection, University of Oradea, 26 Gen. Magheru St., 410087 Oradea, Romania; otirpe@uoradea.ro

**Keywords:** consumer behavior, food habits, COVID-19, crises period

## Abstract

Crisis periods such as the COVID-19 pandemic may reshape consumers’ behavior and challenge all food chain actors on how to assure and better respond to consumers’ needs and wants. This study aimed to reveal the main concerns of consumers related to food consumption during the COVID-19 pandemic and to identify factors that may influence their behavior. An online survey was performed among 859 Romanian consumers. The Principal Component Analysis revealed five factors: ecofriendly, socio-economic aspects, food waste, plant-based food, and easily accessible food, which affected consumers’ food behavior during the COVID-19 pandemic. It was noticed that females tended to be more preoccupied with the socio-economic aspects and food waste components, compared to males. At the same time, older people were more concerned about the ecofriendly, socio-economic aspects and health concerns, compared with the younger group, the differences being statistically significant. These insights provide information on crucial aspects that shape consumers’ behavior during crisis periods.

## 1. Introduction

Food choices are more and more complex, since there is a wide variety of products on one hand, and consumers are being more informed and preoccupied about their health and a healthy lifestyle on the other hand [1]. Several factors influencing food consumers’ behavior were identified such as health, natural content, taste preferences, food attitudes, budget, price, and household structure [2,3,4,5,6,7].

During the last two years, the COVID-19 pandemic has had a significant influence on people all across the world due to two factors: dread of disease and social isolation. As a response to environmental changes, these two factors were able to alter consumer behavior [8,9,10,11] and provide them with a new viewpoint on life [12]. Consumers were confronted with a severe crisis that affected all parts of their lives: social, economic, and psychological, with two elements influencing their behavior: risk attitude and risk perception [13]. Previous crises assisted researchers in better understanding the changes that occur for individuals, thus Flatters and Willmott emphasized the most essential ones, such as demand simplification, recycling emphasis, and the need for simplicity across value-oriented items [14]. Since the pandemic’s long-term crisis has resulted in permanent alterations, researchers believe the effects will be worldwide and long-term, with a direct impact on human society [15]. Consumer behavior was shaped by the COVID-19 crisis positively, by paying more attention to prices, but also valorizing more the eco-friendly and socially responsible companies, paying more attention to the environmental impact of their actions [16], shopping habits, and food waste management [17,18].

It has been noticed that the shopping habits changed substantially, or new ones emerged, such as online shopping, home deliveries, and cashless payments [19]. The changes brought by the pandemic refer to the reduced frequency of shopping, increasing the time allocated to the decision-making process and focusing mainly on the essential goods [20]. The changing patterns in shopping habits are supported by studies, with cross-cultural research obtaining interesting results, where more than half of the respondents from 13 countries declared that their current shopping habits will be maintained completely in the post-pandemic era [21].

The main issue that has to be addressed in this context is to understand the consumers’ concerns regarding food consumption during the COVID-19 pandemic and identify factors that may influence their behavior in crisis periods so that companies could adapt their marketing and management strategies to meet the newly developed needs and desires. The study also focused on understanding the relationship between the identified factors and the socio-demographic characteristics of the respondents. Apart from the already investigated socio-economical concerns regarding food purchase during the pandemic, the present study outlines the importance of sustainable food consumption during a crisis period and the increased awareness of the impact caused by consumers’ shopping habits on the environment.

## 2. Literature Review on Food Choice for Consumption during COVID-19

The COVID-19 pandemic has undoubtedly impacted the lives of people all around the globe, with significant changes in a large number of both personal and professional aspects, with implications for environmentally conscious consumer behavior, environmental sustainability, social responsibility and human well-being [22,23]. The changes occurred due to a disruption in the supply of critical commodities, as well as other concerns among the consumers [24,25].

Research has also shown that the adoption of an eco-label showing a product’s environmental footprint enables supermarket customers to make informed but time-saving decisions, even for customers for which sustainability did not represent a priority in this process [23,26]. Marketing strategies, particularly communication strategies, can play a significant role in customers’ education on environmental knowledge, as well as general environmental awareness [27]. The ecological market segment is based on environmental patterns and self-fulfillment values, with customers interested in companies that are environmentally conscious [28], together with the association of pro-environmental desire and consumers’ eco-friendly activity intentions, mediated by psychological distance, competence, relatedness, and fulfillment needs [29,30]. Additionally, a prosocial attitude and green values also impact green purchasing behavior, a fact emphasized by the increasing evidence of negative environmental changes due to human activity [31,32].

Moreover, consumers’ desire to eat seasonal fruits and vegetables was driven by taste and environmental factors, whereas health-related and ethical motives drove their willingness to limit meat consumption and willingness to pay more for environmentally friendly products [33]. Women and consumers who favor natural foods were more likely to embrace environmentally friendly eating habits and consumer behavior changes to prevent and limit food waste [22,32,34].

Customers’ shopping habits and attitudes concerning grocery shopping and cooking changed during the COVID-19 pandemic [35]. The increase in the frequency and diversity of cooking is associated with a reduced quantity of food waste during the lockdown [36]. There is also a greater inclination for eating more sustainable food at home, but not when dining out, with consumers also stating that food safety and hygiene are more important to them [37]. Consumers prioritize meals and fresh product preparation by reducing their market visits in favor of delivery services and online purchasing platforms and prefer cost-effective packaging and products [38]. Online purchases and curbside pickup became more popular during this period [39]. Moreover, during the lockdown people would rather shop from nearby places and local producers and choose higher-quality goods, instead of crowded supermarkets [40,41,42].

Due to the COVID-19 pandemic, changes occurred also in purchasing power. Anxieties about one’s health and fears about one’s financial situation have an impact on buying behavior, furthermore, demographic factors such as age, gender, race, wealth, and marital status have a substantial impact on their purchasing preferences [39,43]. Country and age may have a significant interaction effect on the individual financial situation, while other factors such as gender, education, and income may differ among consumers with low, medium, and high crisis perception [44]. Hasan et al. emphasized that customers with a higher crisis perception reported more changes in their purchasing behavior in relationship to the pandemic [44].

When it comes to consumer and purchase habits, the pandemic was similar in terms of increased quantities of food, hygiene products, various medicines being acquired, to past disruptive events causing similar psychological stress, requiring proper adaptation [45,46]. This has further increased the purchase of canned and other low perishable goods [45,46]. In Italy, just before lockdown, the demand for sanitizing alcohol and hygiene products significantly increased, while raw materials and products with a long shelf-life such as rice, pasta, flour and frozen foods have also seen an important rise in the preferences of Italians [47]. Chenarides et al. analyzed how this behavior changed in consumers from two American states, by comparing shopping habits from before and during the pandemic and found that more than half of respondents did not change their diet and only 9% affirmed a healthier diet [41]. Furthermore, females consumed less meat than males, while households with children sought to buy more fresh products [41]. In terms of income, people with lower incomes reported cutbacks on fresh produces and regardless of the latest form of education accomplished, the quantity of frozen foods purchased decreased significantly [41]. Janssen et al. illustrated similar findings regarding changes in consumers’ behavior, establishing a correlation between a lower quantity of fresh products being purchased and decreased household income due to the pandemic [48]. Additionally, the study found that households with children were more likely to increase the consumption of fresh fruits and vegetables, and in general women were more likely to consume a larger quantity of fruits and vegetables than men [48].

The changes in food consumption behavior during the pandemic led in many cases to food waste reduction, often due to the concern for future resupply and increased spending rather than environmental interests [49,50]. Fanelli emphasized that Italian consumers increased the quantity of fresh groceries purchased and chose neighborhood shops and local producers in the detriment of supermarkets, but their shopping was based on shopping lists [49]. The new habit of consulting the shopping list and reusing the food scraps to a greater extent leads to a more organized and responsible consumption behavior. In contrast, Filimonau et al. reported an increased food waste in English households, mainly due to delivery and takeaway food ordering and overcooking [51].

Worldwide, consumers’ behavior has undergone significant changes in terms of food consumption, strongly influenced by various socio-demographic characteristics of the consumers. Current existing research assessed the changes in consumers’ behavior due to the COVID-19 pandemic, focusing mainly on how the pandemic determined a series of changes, including dietary ones, which further translated into shopping behavior changes. Additionally, knowledge gap was observed between studies analyzing how consumers perceive food waste during crisis periods, with research focusing on the socio-economical aspects of food purchasing and studies assessing the impact of consumers’ behavior on the environment. Thus, in correlation with the existing research, a better understanding of this phenomenon is needed to fully assess the consumers’ decisions regarding food purchases, attitude toward the environment, and food waste. Moreover, the extent to which the socio-demographic characteristics of the consumers influence the process of decision-making when it comes to food consumption is of great importance. Thus, the research questions that arise are: What are the main concerns of consumers related to food during crisis periods? Do the identified concern factors differ among consumers along socio-demographic dimensions?

## 3. Materials and Methods

The research was conducted among residents older than 18 years from the North-West Development Region of Romania during May-October 2020, to identify consumers’ concerns regarding food consumption during a crisis period. The research consisted of two main steps: first, an analysis of the thematic bibliography was conducted, followed by a primary data collection. An online survey based on a questionnaire was carried out, and in the end a total number of 859 responses were validated. The collected data were divided into two main sections: (i) socio-demographic characteristics (gender, age, education level, monthly net household income, children in the household, place of residency) and (ii): factors that affect consumer behavior during crises period (a set of 22 items evaluated on scale Likert scale type, from little extend to very large extend). A pilot test was conducted to check the reliability of the research instrument. Chronbach’s alpha was 0.915, indicating that the scale used during this study is reliable [52]. Harman’s single-factor test was employed to verify the presence of common method bias [53]. The first single factor in the unrotated factor matrix explained 38.3% of the variance, below the suggested 50% threshold.

Descriptive statistics were used to describe the profile of the respondents. The results revealed that 61.1% of the total number of participants in the study were female, while 38.9% were male. Furthermore, more than half of the respondents (53.1%) were under the age of 40, while 46.9% were above the age of 40. When it comes to education, the vast majority of the participants (74%) held a university degree, with only 26% having graduated from high school or less, among which only 2.8% had completed less than eight classes. In terms of household income, more than half of the participants (56.1%) stated a monthly net household income of over 4200 RON, while 43.9 percent stated a monthly net household income of less than 4200 RON. 51.3 percent of respondents had children under the age of 18 living in the same home, while 48.7% did not. Regarding their place of residence, the vast majority of the participants (73.9%) were living in urban areas, while 26.1% lived in rural regions (Table 1).

Furthermore, the 22 variables were factor-analyzed using Principal Component Analysis with the Varimax rotation. Factors with an eigenvalue higher than 1 and factor loading equal to or higher than 0.4 were considered significant and included in the analysis [52,54]. The non-parametric Mann-Whitney U was used to find out if there were any significant differences in socio-demographic groups. A *p*-value of less than 0.05 was considered statistically significant.

## 4. Results

After the principal component analysis was run, a significant Barlett’s test of sphericity (Chi-Square = 9369.641; *p* < 0.000), with a Kaiser-Meyer-Olkin (KMO) measure of sampling of 0.922, above the critical value of 0.6, indicating that data are suitable for the principal component analysis [52]. The PCA of the 22 variables using Varimax rotation yielded a five-component solution, explaining 64.741% of the total variance. Factors with eigenvalues larger than one were chosen and evaluated. Moreover, the Cronbach’s alpha reliability coefficient was used to assess the internal consistency of each component, returning a significant value of 0.915. The five components of the PCA, together with their corresponding variables, are presented in Table 2.

Accounting for 38.36% of the total variance and having a reliability coefficient of 0.896, the “Ecofriendly” component analyzed consumers’ perception towards sustainability, local products, recycling and overall environmental safety, in relation to their shopping habits. It had a mean of 3.52 and SD of 0.834, with respondents being inclined to purchase only products they strictly need (3.78 ± 0.998), to shop more often from local producers (3.69 ± 1.048) and choose natural products (3.69 ± 1.013). Additionally, consumers also showed a tendency to recycle more (3.53 ± 1.076) and choose certain products with recyclable packaging (3.39 ± 1.053). Out of the eight variables included in this component, participants were least concerned about the food production process (3.29 ± 1.125) and whether it was safely obtained from the environment (3.35 ± 1.047).

The second component “Socio-economic aspects” explained 9.14% of the variance and groups 7 items, with a reliability coefficient of 0.841. The results indicated that the respondents were inclined to choose healthier products (3.9 ± 0.914), being significantly concerned about their health (3.78 ± 1.041). Consumers did, however, pay close attention to their financial status and safety (3.72 ± 0.995), as well as food prices (3.66 ± 0.985), while taking an important interest in reducing food waste (3.65 ± 1.009). Participants were, on the other hand, less likely to only pick items from certain brands (3.38 ± 1.038) and were less impacted by social distancing (3.28 ± 1.180).

The third component “Food waste” explained 7.65% of the variance and had a reliability coefficient of 0.796. In terms of consumer attitudes around food waste, respondents were more likely to organize their shopping lists more carefully (3.55 ± 1.1) and reduce the amount of food that is thrown away (3.46 ± 1.194), while also sticking to their shopping list (3.46 ± 1.054).

The “Plant-based food” component accounted for 4.99% of the total variance, with a mean of 2.57 ± 0.986, while the reliability coefficient was 0.771. Participants were also concerned about their health, with the majority of them buying bio-certified products (2.72 ± 1.085) and, to a lesser extent, only products of plant origin (2.43 ± 1.101).

Regarding the “Easily accessible food” component, it represented 4.58% of the total variance and had a mean of 2.42, SD of 0.894, and reliability coefficient of 0.704. Thus, consumers declared purchasing a higher quantity of canned products (2.51 ± 1.093), while also buying products that can be quickly cooked (2.36 ± 1.145). Moreover, the respondents were less likely to be influenced by online food advertising (2.4 ± 1.129).

Table 3 illustrates the relationship between the socio-demographic characteristics of the respondents and the five components resulting from the PCA. Significant differences between males and females were observed in the socio-economic (*p* < 0.000) and food waste (*p* = 0.001) components, with females paying more attention to both of them (3.69 ± 0.69 and 3.57 ± 0.92, respectively).

When it comes to age, people over 40 years of age were more considerate of the impact their shopping habits have on the environment, taking more interest in the “Ecofriendly” component (3.65 ± 0.79), compared to younger respondents (*p* = 0.000). Additionally, they were more concerned about socio-economic (*p* = 0.002; 3.72 ± 0.72) and health aspects (*p* = 0.003; 2.68 ± 0.98) as well.

Notable differences have also been observed regarding the education level of the respondents, with consumers with a university degree paying more attention to food waste than respondents with a lower form of education (*p* = 0.008; 3.55 ± 0.89). Nevertheless, no significant distinctions have been observed in relation to the monthly net household income of the respondents.

Moreover, consumers took a different interest in the “Ecofriendly” component, with respondents living with their children in the household (*p* = 0.043; 3.57 ± 0.86) and those residing in rural areas (*p* = 0.019; 3.61 ± 0.88) being more preoccupied with sustainable food shopping and recycling.

## 5. Discussion

The study aimed to assess the concerns regarding food consumption during the COVID-19 pandemic, as well as to identify factors influencing consumers’ behavior during periods of crisis. Our research has underlined the fact that during the COVID-19 pandemic, people were more conscious about the environment and tried to adopt a more sustainable behavior regarding food purchase and consumption decisions. This has mainly been expressed by choosing products with recyclable packaging, buying natural products and from local producers, while also minimizing food losses in the household, females tend to be more aware of the food waste management and socio-economic aspects, compared to males. These findings have previously been confirmed in studies conducted in other countries, such as Italy [49,50] and Tunisia [55].

Nevertheless, health concerns due to the pandemic might have influenced certain behaviors, such as purchasing products from local, nearby producers to limit the exposure and avoid crowded places [40,41]. Older people and families with children pay more attention to the “ecofriendly” factor compared with younger people and families without children (*p* < 0.05), a fact that might be due to a higher preoccupation for health among these two categories of consumers (mature consumers and children). Additionally, the lockdown, closing of restaurants and working from home has urged people to cook more often or order take-away. Regarding the “easily accessible food” the respondents did not tend to consume more than in the period previous to the pandemic, although, at the beginning of the pandemic and lockdown, studies reported an increased quantity of “comfort foods” such as snacks and sweets being purchased, this trend has seen gradual decline afterward [41,56]. Furthermore, not only did people increase their food products purchases, but rather searched for fresh and higher quality goods [56]. This fact translated into a growing interest in bio-certified products or plant-based foods, a phenomenon also observed by Eftimov et al. [57], however, this trend was not confirmed within this study. No significant changes were found in terms of buying plant origin products and bio-certified plant products.

When it comes to the type of products preferred by consumers, a certain universal pattern has yet to be determined, with some studies reporting healthier behaviors [58], while others showed an increase in the quantities of snacks and frozen foods [48,59] or, in some cases, no changes in the diet at all [41]. Several factors should be considered regarding these changes, such as income and education level, some studies report that unhealthier and lesser quality food is being purchased by lower-income consumers [56].

Moreover, people feared future economic recession due to the pandemic, which has led to socio-economic concerns reflected in changes in food purchasing behavior [12,56]. While looking to find better ways to manage their finances, consumers paid more attention to food prices, as well as purchasing healthier products. Our study concluded that females were more preoccupied with this fact than males, while older participants paid more attention to these socio-economic aspects than younger ones.

Among other changes in consumers’ habits during the pandemic, food waste represents an important aspect taken into account by more and more consumers [50,58,60]. This study also revealed that females and respondents with a university degree were more interested in reducing food waste.

Interestingly, it appeared that easily accessible food does not appeal to the analyzed Romanian consumers as much as the other components do, regardless of the socio-demographic characteristics of the respondents. Consumers showed low interest in canned products or foods that are quick and easy to cook and are rather impassible to online food advertising. It is worth mentioning that, at the beginning of the pandemic, growing insecurities regarding food safety have prompted many people to stockpile goods, especially products with long shelf life, a phenomenon observed in many countries around the globe [45,46,47,56].

## 6. Conclusions

It is becoming ever clearer that the pandemic has greatly influenced a significant number of aspects of our everyday lives, bringing along changes in our behavior that are going to stay. A large number of studies have shown a shift in the consumers’ preferences and choices when it comes to food consumption, as well as cooking meals at home and dining out. Moreover, it appeared that the pandemic has made people more conscious regarding food waste, the majority of the research in this domain shows a significant reduction in the quantity of food thrown out and the development of alternative and secondary ways of using the leftovers. Regardless of the reason, whether it is represented by economical or environmental concerns, this positive trend should be encouraged as a more sustainable way of food consumption. The study revealed that people with higher education are more likely to pay more attention to food waste.

Additionally, the results showed a growing interest in environmentally friendly and natural products, with food consumption behavior also being influenced by health and socio-economic factors. People residing in rural areas were more attentive to recycling and sustainable food shopping, while older respondents were more concerned about the impact of products on their health and the impact their choices might have on the environment.

The main contribution of this research consists in revealing how the pandemic has changed consumers’ food behavior. Consumers are more preoccupied with socio-economic aspects and how to reduce the quantity of thrown food, especially in the case of the more educated who became more aware of food waste. The results confirm that under crisis periods the consumers’ behavior is changing, and the producers need to adapt their products to the new trend.

When it comes to the limitations of the study, the questionnaire included a significant number of participants with a higher education (i.e., university degree or higher), which might not be entirely representative for the entire Romanian population and focused on one development region. Further research may deepen the findings of our research by extending the research area and assuring a better representation of consumers pertaining to all education groups.

## Figures and Tables

**Table 1:** Respondents’ socio-demographic profile.

Characteristics		Sample (%)
Gender	Female	61.1
	Male	38.9
Age	18–39 years	53.1
	>40 years	46.9
Education level	High school or less *	26.0
	University degree	74.0
Monthly net household income	≤4200 RON	43.9
	>4200 RON	56.1
Children in the household (<18 years)	No	48.7
	Yes	51.3
Place of residency	Rural	26.1
	Urban	73.9

* Only 2.8% declared less than 8 classes as the level of education.

**Table 2:** Principal component analysis.

Eigenvalue	Variance %	Factor	Item	Factor Loading	Mean	SD
80.43	38.36	Ecofriendly Mean = 3.52 ± 0.834 α = 0.896	I choose products with recyclable packaging more often	0.841	3.39	1.053
			I recycle more	0.782	3.53	1.076
			I buy natural products more often	0.776	3.69	1.013
			I am more concerned about the food production process	0.707	3.29	1.125
			I choose more often food products obtained safely for the environment	0.692	3.35	1.047
			I buy food more often from local producers	0.663	3.69	1.048
			I only buy products I really need	0.551	3.78	0.998
20.01	9.014	Socio-economic aspects Mean = 3.63 ± 0.736 α = 0.841	I am concerned about the financial situation/safety	0.762	3.72	0.995
			I am more concerned about my health	0.702	3.78	1.041
			I pay more attention to food prices	0.693	3.66	0.985
			I am affected by social distancing	0.626	3.28	1.180
			I choose healthier products	0.585	3.90	0.914
			I am concerned about reducing food waste	0.558	3.65	1.009
			I only buy products belonging to known brands	0.550	3.38	1.038
10.68	7.065	Food waste 3.48 ± 0.942 α = 0.796	I plan my shopping list more carefully	0.764	3.55	1.100
			I tend to stick to my shopping list	0.734	3.46	1.054
			I throw out a lower quantity of food	0.718	3.46	1.194
10.09	4.099	Plant-based food 2.57 ± 0.986 α = 0.771	I only buy products of plant origin	0.796	2.43	1.101
			I only buy bio-certified plant-based products	0.777	2.72	1.085
10.00	4.058	Easily accessible food 2.42 ± 0.894 α = 0.706	I buy more canned products	0.827	2.51	1.093
			I buy products that cook quickly	0.789	2.36	1.145
			I am influenced by online food advertising	0.592	2.40	1.129
Total variance %	62.596 α = 0.915					

**Table 3:** Relationship between the socio-demographic characteristics and PCA components.

Characteristics		Ecofriendly	Socio-Economic Aspects	Food Waste	Plant-Based Food	Easily Accessible Food
Gender	Female	3.55 (0.83)	3.69 (0.69)	3.57 (0.92)	2.57 (0.98)	2.39 (0.88)
	Male	3.47 (0.84)	3.52 (0.78)	3.36 (0.97)	2.57 (0.97)	2.49 (0.92)
	*p*-value	0.156	0.000 ***	0.001 ***	0.755	0.074
Age	18–39 years	3.40 (0.85)	3.54 (0.74)	3.44 (0.94)	2.47 (0.97)	2.46 (0.89)
	>40 years	3.65 (0.79)	3.72 (0.72)	3.54 (0.94)	2.68 (0.98)	2.39 (0.89)
	*p*-value	0.000 ***	0.002 **	0.144	0.003 **	0.124
Education level	High school or less	3.41 (0.94)	3.53 (0.89)	3.31 (1.06)	2.58 (1.04)	2.49 (0.98)
	University degree	3.56 (0.79)	3.66 (0.67)	3.55 (0.89)	2.58 (0.96)	2.40 (0.86)
	*p*-value	0.066	0.167	0.008 **	0.959	0.315
Monthly net household income	≤4200 RON	3.49 (0.90)	3.61 (0.81)	3.45 (1.01)	2.58 (1.03)	2.47 (0.95)
	>4200 RON	3.54 (0.78)	3.64 (0.68)	3.52 (0.88)	2.56 (0.95)	2.39 (0.85)
	*p*-value	0.673	0.663	0.625	0.401	0.511
Children in the household (<18 years)	No	3.47 (0.81)	3.60 (0.68)	3.48 (0.89)	2.52 (0.95)	2.38 (0.84)
	Yes	3.57 (0.86)	3.65 (0.78)	3.49 (0.98)	2.62 (1.02)	2.47 (0.94)
	*p*-value	0.043*	0.077	0.471	0.329	0.306
Place of residency	Rural	3.61 (0.88)	3.64 (0.77)	3.47 (0.98)	2.67 (1.02)	2.52 (0.96)
	Urban	3.49 (0.82)	3.63 (0.72)	3.49 (0.93)	2.54 (0.97)	2.39 (0.86)
	p-value	0.019 *	0.365	0.745	0.100	0.093

* significant at 5% level, ** significant at 1% level, *** significant at 0.1% level.

## Data Availability

Data is contained within the article.
